# Anencephaly: Do the Pregnancy and Maternal Characteristics Impact the Pregnancy Outcome?

**DOI:** 10.5402/2012/127490

**Published:** 2012-01-19

**Authors:** Isabela Nelly Machado, Sílvia Dante Martinez, Ricardo Barini

**Affiliations:** ^1^Fetal Medicine Program, Department of Obstetrics and Gynecology, School of Medicine, Universidade Estadual de Campinas (UNICAMP), Campinas, SP, Brazil; ^2^Department of Obstetrics and Gynecology, UNICAMP, Rua Alexander Fleming, 101, Cidade Universitária, 13083-970 Campinas, SP, Brazil

## Abstract

*Objective*. To describe the characteristics of obstetric and perinatal outcome of a group of pregnancies complicated by an anencephalic fetus. *Methods*. Observational study including anencephalic fetuses, divided into groups according to the evolution of pregnancy: elective termination of pregnancy ETP; stillbirths (SBs); live births (LBs), and loss of follow-up. After a univariate description of the sample, some variables were compared using statistical tests. *Results*. 180 anencephalic fetuses were included. The mean maternal age was 25.3 years. In 71 fetuses (39%) were found additional anomalies. Comparing the groups, no statistical differences in maternal age (*P* = 0.5315), parity (*P* = 0.6070), number of previous abortion (*P* = 0.7464), fetal sex (*P* = 0.0502) and additional anomalies (*P* = 0.186) were found. Among those fetuses whose parents opted for continuation of pregnancy (*n* = 53), 20 spontaneous intrauterine deaths occurred (38%) and 33 were live births (62%). The average postnatal survival time was 51 minutes. There was no association between survival time and gestational age (*P* = 0.6125) or the presence of additional malformations (*P* = 0.1948). *Conclusion*. Results presented here could contribute to a better understanding of the natural history of this malformation, allowing obstetricians a more detailed discussion with the families.

## 1. Introduction

Anencephaly is a neural tube defect (NTD) caused by a failure of closure in the cranial neuropore between the third and fourth week of gestation (23rd and 26th embryonic day), resulting in the absence of a major portion of the brain, skull, and scalp [1]. The brain lacks part or the entire cerebrum, and the remaining brain tissue is often exposed to injury from amniotic fluid. The precise etiology of anencephaly and other NTDs is unknown; however, socioeconomic status, environmental conditions, and the genetics of both population and familial ancestry are indicated [2–4]. The prenatal detection of anencephaly through the antenatal ultrasound is virtually 100% [5].

After the periconceptional folic acid supplementation, the prevalence of anencephaly-affected pregnancies declined worldwide, but no significant change in prevalence was observed in the subsequent years [6, 7]. Its actual prevalence is estimated in 1/1000 pregnancies [8], but because of the option of pregnancy termination after prenatal diagnosis, the exact incidence is not easily accessed.

Anencephalic newborns are not viable or treatable, and so, it is classified as a lethal neural tube defect. Although stillbirth is a common outcome of fetal anencephaly, some affected fetuses are born alive with a rudimentary brain. Lacking a functioning cerebrum, they are incapable of consciousness and of experiencing pain, although the brain stem may support reflex actions such as breathing, and occasionally responses to sound or touch [8]. Some rudimentary lower brain development is usually present with a small proportion of neonates surviving a few days; however, the timing of death shows marked variation in published records.

The aim of this study was to describe the obstetric and perinatal outcome of a group of pregnancies complicated by an anencephalic fetus, in an attempt to provide obstetricians important tools for a more detailed discussion about prenatal management with the affected families.

## 2. Materials and Methods

A retrospective observational study was conducted by analyzing medical records of fetuses with a diagnosis of anencephaly through antenatal ultrasound and who were referred to the Fetal Medicine Program of the Women's Hospital Professor Dr. Jose Aristodeno Pinotti (CAISM/UNICAMP), from August 2000 to July 2010, after the protocol approval by the institution's ethical committee. Anencephalic fetuses with aneuploidy on G-banded karyotype were excluded.

Clinical data were obtained from the medical records. Besides demographic characterization, it included the complete findings from antenatal ultrasound recorded, the babies' features observed through clinical examination by neonatologists and geneticists after birth, and necropsy findings. Previous miscarriages were considered when there was the clinical confirmation of pregnancy that did not develop.

The fetuses were divided into groups according to the evolution of pregnancy: elective termination of pregnancy ETPs, stillbirths (SBs), live births (LBs), and loss of follow-up. Perinatal variables included prenatal complications (twinning, polyhydramnios, others), pregnancy duration, delivery route, birth weight, the Apgar score, survival time, associated malformations and maternal postnatal complications. Associated malformation was considered when at least one other anomaly not included into the tube neural defects was present in the fetus. Polyhydramnios was defined as the ultrasound estimation of amniotic fluid index >20 cm and premature delivery as the delivery occurring before 37 weeks of gestation. For gestational age, the complete weeks of gestation were considered.

For the outcome variables analysis, the cases without follow-up records were excluded. After a univariate description of the sample, some variables were compared between groups using statistical tests (software SAS version 9.2).

## 3. Results

**Figure 1:**
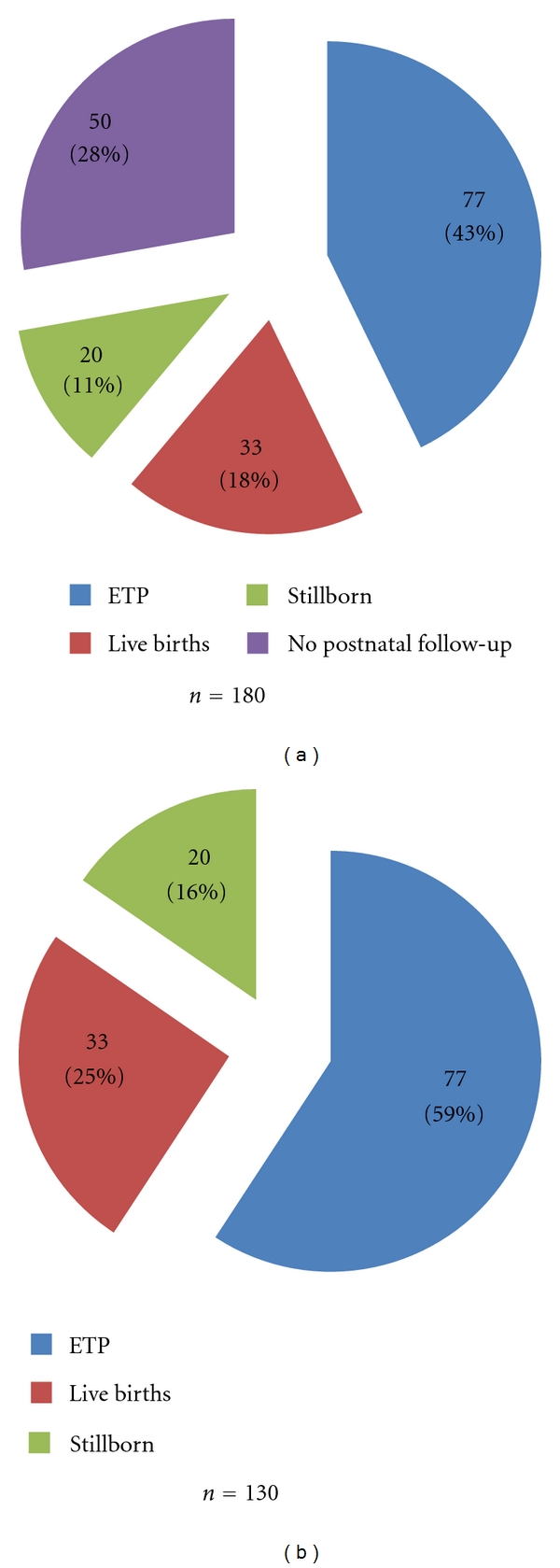
Frequency distribution of the anencephalic fetuses according to the outcome. ETP: elective terminated pregnancies. *n* = 180 (all included), *n* = 130 (excluding the cases without postnatal follow-up).

One hundred and eighty cases of anencephaly were included in this study. It was observed that 77 of the parents (43%) chose not to continue the pregnancy. Fifty families did not continue the prenatal management in our tertiary service after the diagnosis confirmation (28%), and we could not check their perinatal data. The distribution of the fetuses according to the outcome groups is shown in Figure 1.

Median and mean maternal age was 25 years (range 14–45), 81% (*n* = 146) were younger than 30 years old, 39% (*n* = 70) were primigravidas, and 45% (*n* = 81) were primiparous. Twenty-seven women (15%) had one previous spontaneous miscarriage, and eight women (4.5%) had at least two previous miscarriages.

There was a female preponderance (64%) among this anencephalic offspring sample. There was no significant difference in the frequencies of maternal age (*P* = 0.5315; chi-Square), gravidity (*P* = 0.3204; chi-Square), parity (*P* = 0.6070; Fisher's exact test), number of miscarriages (*P* = 0.7464; Fisher's exact test), and fetal gender (*P* = 0.0502; Fisher's exact test) between the four outcome groups.

Seven anencephalic fetuses were from twin pregnancies (4%), one of them from a triplet pregnancy. For one case, there was no postnatal available data. In 4 of the remaining 6 cases, both fetuses were live births, and in 2 pairs, the anencephalic fetuses were stillbirths. In one of these stillbirth cases, the second twin had bilateral renal agenesis and also died *in utero*. All other siblings' twins showed no morphological abnormalities and were live births, including the triplet pregnancy case.

One hundred and seventeen fetuses delivered by vaginal root (117/130; 90%). Among the thirteen cesarean deliveries, two occurred in other services and we were not informed about the indications. Three cesarean deliveries occurred in the elective termination of the pregnancy group, 1 due to failure of the labor induction and 2 because women had two previous cesarean sections. The remaining 8 cesarean deliveries occurred into the live birth group, 6 due to prior cesarean sections, 1 occurred in the triplet pregnancy, and 1 case had no indication in the medical reports to be found. There were no maternal postnatal complications.

Twenty-seven pregnancies (27/180; 15%) developed polyhydramnios. In seventy-one fetuses (71/180; 39%), the anencephaly was associated to at least one other anomaly not included into the tube neural defects. The frequency of the associated malformations is listed in Table 1. In the group of 20 anencephalic stillbirths, 9 fetuses (45%) presented with associated malformation and in 11 fetuses (55%) the anencephaly was isolated. There was no significant difference in the frequency of associated malformation between the groups of life birth and stillbirth (*P* = 0.186; chi-Square).

Among fetuses whose families decided to continue the pregnancy (*n* = 53), 33 infants were alive at birth (62%) and 20 fetuses died spontaneously *in utero* (38%). The mean and median gestational ages for the stillborn deliveries were 31 weeks (range 19–41) and for the live birth deliveries were 32 weeks (range 25–43). The mean weight at birth was 1360 g (range 500 g–2810 g) for the live births and 1250 g (range 130 g–2800 g) for the stillbirths. The postnatal survival time ranged from 1 minute to 48 hours (median survival of 51 minutes). Of the 33 live-born infants, 31 (94%) died within 24 hours and 22 (67%) of them within the first hour.

Among the 27 live birth fetuses who survived more than 5 minutes, the median Apgar score at the 5th minute was 1. There was no correlation between the survival time and the gestational age (*P* = 0.6125; Spearman's correlation coefficient). Also, there was no correlation between the survival time and the presence of associated malformations (*P* = 0.1948; Mann-Whitney test). The frequency of premature, term, and after-term deliveries among the stillborn and live-born is presented in Table 2.

In the group of 77 elective terminated pregnancies, the mean and median gestational ages at the delivering time were 23 weeks (range 14–36), and the mean weight at birth was 667 g (range 54 g–1870 g). Comparing the perinatal variables between the four outcome groups, the gestational age at delivery and birth weight were the only ones with significant difference (*P* < 0.0001 for both, the Kruskal-Wallis test). This difference was observed when we compared the elective terminated pregnancy group with the stillborn group and with the live-birth group (Table 3).

## 4. Discussion

This study evaluated the development of 180 fetuses from diagnosis to the outcome of pregnancies in a period of 10 years and represents the largest cohort of anencephaly cases from a unique medical service.

The strength of this study, besides the large number of included cases, is that we not only considered only the phenotype definition based on the ultrasound records, which cannot always reflect the complete fetus phenotype, but also we confirmed the prenatally diagnosed malformations with postnatal or *postmortem* evaluation, giving a detailed morphological characterization of the cases. This more detailed evaluation is essential for a definitive diagnosis and etiological establishment [4]. Also, our data could determine the timing of death, contributing to the prognosis discussion with affected families.

The percentage of cases with failure to achieve postnatal follow-up in our series was 28% (50 cases). It does not invalidate our results, since there were no differences between groups regarding maternal parameters. In fact, this rate corresponds to the large number of families that seek our tertiary health center just to confirm the anencephaly diagnosis, often in the hope of finding a more favorable prognosis for the offspring.

Our data could confirm several previous reports. The young maternal age, half of them primiparous, have already been observed [12]. Indeed, having had less than three gestations was appointed as a risk factor for NTDs in a sample of 89 affected live born infants [9]. The female preponderance in our anencephalic offspring sample was also reported previously [10, 13], although a recent study did not reveal this preponderance [12].

No association was found regarding maternal age, gravidity, parity, and number of miscarriages among the outcome groups, showing that these were not factors associated with the decision of couples regarding the maintenance of pregnancies or with the final outcome of cases.

Four percent of the pregnancies were complicated by twinning in our series, one of them with both fetuses affected. This complication was found in 10% of 211 pregnancies according to the information collected from a website, all cases with only one fetus affected [10]. This research also observed one triplet affected pregnancy with one anencephalic fetus, like in our sample. As well recorded previously, polyhydramnios was a common prenatal finding, complicating 15% of our cases, lower than the 27% prevalence recorded in other series [10, 12]. It is appointed as a risk factor for preterm deliveries and maternal respiratory distress and may be discussed with the pregnant women.

The prevalence of additional malformations not included into the tube neural defects was 39% in this study, in contrast with 8% in the parental reports to a website [10]. It could be explained by our data collection methodology, which included a detailed study of the fetuses and newborns morphology. In a series of 24 fetuses with anencephaly among a NTD sample [9] other anomalies were identified in 13 fetuses (54%). In a recent series including the postmortem analysis of 14 anencephalic fetuses, additional anomalies were found in 5 fetuses (25.7%) [4]. The observed prevalence of additional malformations in our series was 45% among stillbirths and 64% among live born infants, but there was no significant difference in the frequency of associated malformation between the two groups, indicating that the presence of additional malformation does not determine the fetal/neonatal death.

In recent years, the abortion response to prenatal detection of anencephaly has come under legal challenge, particularly in South America (notably in Argentina and Brazil) and Ireland, probably related to religious reasons [8]. In the present study, conducted in Brazil, where women are required to obtain judicial approval before physicians will terminate their pregnancies, 43% of the families chose not to continue the pregnancy. In the UK, between 1980 and 2007, more than 90% of the anencephalic cases underwent termination of pregnancy [14], showing cultural and legal differences among different countries. It is not our intention to discuss ethical, legal, and moral aspects involved in elective termination of anencephaly-affected pregnancies, but rather to describe and discuss the pregnancies outcome.

The gestational age at delivery and birth weight were the only variables that showed statistical significance in comparing all the perinatal variables between the outcome groups. These differences were observed when we compared the elective terminated pregnancy group with the stillborn group and with the live birth group. It means that elective termination of pregnancy has significant impact on length of gestation and, therefore, reduces the birth weight.

Vaginal delivery was performed in 90% (117/130) of the pregnancies, in accordance with our health service protocol in which this is the first choice for the delivery route in the cases with poor prognosis. Although we could access clear indications in 3 instances, most of the thirteen cesarean deliveries occurred due to previous cesarean sections (8/13).

Among the sample of 53 fetuses whose families decided to continue the pregnancy, the spontaneous prenatal death *in utero* occurred in 20 fetuses (38%), against the prevalence of 33 live infants (62%), consistent with other series which showed that the majority of (or at least half of them) the anencephalic fetuses die after birth and not *in utero* (Table 4). This finding is in contrast with a 42% of survival in a study that considered only the anencephaly as an isolated abnormality [12], but, it is important to emphasize that, even in the stillborn group, most deaths tend to occur during labor, not before it [10, 12]. We did not record the timing of death for the stillbirth fetuses.

Premature delivery (before 37 weeks of gestation) was the most common pregnancies outcome for the 53 fetuses, with a prevalence of 83% (44/53) both for the stillborns and live birth fetuses in our series. The parents in Jaquier et al. [10] had described a prevalence of 34% of prematurity, discordant with our data. The mean gestational age at delivery was 32 weeks (range 19–43), 3 weeks less than that reported by Obeidi et al. [12]. Beside the prematurity, we could observe 3% of postdate gestational periods, also mentioned by others [10].

The median postnatal survival time was 51 minutes, in agreement with the finding of 55 minutes previously registered [12], highlighting the fact that the majority of deaths occurred within the first day (94% in our study and 67% in [10]). We could not find correlation between the survival time neither with the gestational age, nor with the presence of associated malformations, suggesting that the fetus would die due to its pathology, more than due to the prematurity or additional abnormalities.

Offering a broad and current series, the results presented here could contribute to a better understanding of the outcome of the pregnancies affected by this lethal malformation, allowing obstetricians a more detailed discussion with the involved families about fetal and maternal complications, associated conditions, and prognosis of fetuses with prenatal diagnosis of anencephaly.

## Figures and Tables

**Table 1:** Frequency distribution of the associated malformations.

Anomaly		*N*	*N* (total)
Facial defects	Clefts Others	10 17	27
Kidneys and urinary tract defects	Renal agenesis Obstructive uropathies Dysplastic kidney	6 6 5	17
Limbs defects			13
Heart defects			10
Genital defects	Penis hypoplasia Cryptochism Scrotum hypoplasia Ambiguous genitalia Hemiuterus	3 2 1 1 1	8
Gastrointestinal tract defects	Intestinal obstruction Imperforated anus Others	2 1 4	7
Single umbilical artery			6
Omphalocele			5
Congenital diaphragmatic hernia			4
Gastrosquisis			3
Pulmonary defects	Pulmonary agenesis Cystic adenomatoid malfunction of the lung	1 1	2

**Table 2:** Frequency distribution of the premature, term, and after-term deliveries among the stillborn and live-born anencephalic fetuses.

GA at delivery	Stillborn		Live born	
	*n*	%	*n*	%
<37 weeks	17	85	27	82
37–42 weeks	3	15	5	15
>42 weeks	0	0	1	3

GA: gestational age.

**Table 3:** Comparison of gestational age and birthweight between the stillbirth, live birth, and terminated pregnancy groups the (Mann-Whitney test).

	GA	BW
	*P* value	*P* value
Live birth × stillbirth	0.7826	0.5758
Live birth × ETP	<0.0001	<0.0001
Stillbirth × ETP	0.0002	0.0039

GA: gestational age; BW: birthweight; ETP: elective terminated pregnancy.

**Table 4:** Spontaneous outcome of anencephalic fetuses in cited studies.

Study	*N*	Stillbirth (*n*/%)	Live birth (*n*/%)
Aguiar et al., 2003 [9]	24	12 (50%)	12 (50%)
Jaquier et al., 2006 [10]	211*	58 (27%)	153 (73%)
Sedano et al., 2008 [11]	14	2 (14%)	12 (86%)
Obeidi et al., 2010 [12]	26**	15 (58%)	11 (42%)
Machado et al. (this study)	53	20 (38%)	33 (62%)

*N*: number of included fetuses

*data from a website, without subsequent confirmation or review by medical reports

**anencephaly as an isolated abnormality.
